# In-vitro characterization and evaluation of innovative surface modifications of zirconia implants for the optimization of hard and soft tissue management

**DOI:** 10.1186/s40729-026-00713-2

**Published:** 2026-09-05

**Authors:** Fabian Angerer, R. Smeets, J. T. Strenge, M. Gosau, E. Bibiza, B. Schick, K. Eisenmenger, J. H Ehlers, A. Henningsen, S. Fuest

**Affiliations:** 1 Department of Oral and Maxillofacial Surgery, University Medical Center Hamburg-Eppendorf, University of Hamburg, Martinistrasse 52, 20246 Hamburg, Germany; 2 Department of Oral and Maxillofacial Surgery, Division of Regenerative Orofacial Medicine, University Medical Center Hamburg-Eppendorf, Martinistrasse 52, 20246 Hamburg, Germany; 3 goodBIONICS Biotechnologie GmbH, Bahnhofstrasse 6, 87665 Mauerstetten, Germany; 4 TITAN PRÄCIS Metallurgie GmbH, Brahmkoppel 1a, 24558 Henstedt-Ulzburg, Germany

**Keywords:** Zirconia implants, Dental implantology, Calcium phosphate-hydroxyapatite, Cell culture, Microscopy

## Abstract

**Purpose:**

This study focuses on the characterization and evaluation of innovative surface modifications of zirconia implants aimed at optimizing hard and soft tissue management.

**Methods:**

The cytocompatibility of newly developed ceramic implant coatings was assessed. Five different surface types were investigated: uncoated zirconia (Zr), zirconia coated with pure titanium (ZrTi), zirconia coated with calcium phosphate (CaP), zirconia coated with hydroxyapatite (HA) and zirconia coated with calcium phosphate-hydroxyapatite (CaP/HA). Cytocompatibility was assessed using L929 mouse fibroblasts and MC3T3 pre-osteoblasts. Testing was performed in both direct and indirect cell contact with the materials. In the direct contact setup, cells were cultured for 24 h on the test surfaces, followed by live/dead staining and fluorescence microscopy. In the indirect setup, material extracts were prepared and cell viability was quantified after 72 h using lactate dehydrogenase (LDH) and cell proliferation (XTT) assays.

**Results:**

All tested surfaces demonstrated good cytocompatibility without significant cytotoxic effects. Supplementary scanning electron microscopy (SEM) and energy-dispersive X-ray spectroscopy (EDS) analyses confirmed the successful application of the coatings and provided detailed insight into surface topography and elemental composition.

**Conclusion:**

Calcium phosphate and hydroxyapatite-coated zirconia implants represent a promising biocompatible alternative to titanium-based surfaces, with the potential to improve tissue integration and long-term clinical outcomes in ceramic dental implantology.

## Background

Dental implants serve as anchoring structures for prosthetic restorations following tooth extraction, with the goal of achieving both functional and aesthetic rehabilitation [1]. They have been successfully utilized in dental tooth replacement for over four and a half decades [2]. Titanium (Ti) remains by far the most used material for this purpose, due to its excellent biocompatibility, corrosion resistance, and mechanical strength, as well as its favorable osseointegration properties [3]. However, one significant drawback of Ti is its grey color, which can lead to noticeable aesthetic impairments, particularly in the anterior region of patients with thin mucosal biotypes [4]. In addition, immunological responses to Ti have been documented. The incidence of inflammation of the implant bed, known as peri-implantitis, is very high and ranges between 10% and 47.1% of the patient population, depending on the study, and between 6.6% and 36.6% of implants, compared to perimucositis (soft tissue infection) 31% to 64.5% in relation to patients and 21.6% to 38% in relation to the implants placed. In the short term, various modifications to the implant surfaces for micro- and macrostructuring, as well as surgical interventions, can now achieve 5-year survival rates of 95% [5, 6]. As a metal-free counterpart to Ti, zirconium (Zr) dioxide-based ceramic implants have emerged in recent years as an attractive alternative in dental implantology [7].

In the 1990s and early 2000s the first studies on novel ceramic implants made from Zr were published [8, 9]. This material offered a low allergenic potential and further advantages including reduced bacterial adhesion and a tooth-like white color that contributes to superior aesthetic outcomes, especially in the anterior maxilla [10]. Approximately 10 years later, first clinical studies appeared, showing high survival rates within the first 5 years [11, 12]. In the past decade, zirconia implants have demonstrated survival rates comparable to those of titanium implants [13].

Most zirconia implant systems are designed as one-piece configurations, in which the implant body and abutment form a monolithic unit [14]. While these one-piece systems eliminate the risk of a subgingival microgap between the implant and abutment - thus reducing bacterial colonization - they also offer limited prosthetic flexibility. Since abutments cannot be exchanged, individualized prosthetic solutions are restricted and corrections to the implant angulation via the suprastructure are only possible to a limited extent [15]. Surface modifications for optimized healing could be even more important for long-term success, especially with single-piece ceramic implants [16].

Such limitations can be mitigated either by precise preoperative planning and accurate surgical execution or by utilizing novel patient-specific ceramic implant designs. The main advantages of the one-piece system lie in its structural simplicity and in the absence of a subgingival interface that could harbor bacteria [17]. In contrast, two-piece implant systems, which represent the current clinical standard, offer greater prosthetic versatility and allow for stress-free, submerged healing with primary wound closure [14].

Despite the increasingly favorable long-term outcomes of zirconia implants in terms of implant survival and complication rates, there remains a need for further optimization [18]. Enhancements may be achieved through modifications to the manufacturing process - such as altering the implant surface microstructure via sandblasting or acid etching - or through innovative coating strategies and surface modifications, including antibacterial coatings or bioactive interfaces [19, 20]. However, due to the inherent properties of ceramic materials, such as those exhibited by Zr, coating and surface modification procedures remain technically challenging. The material’s smooth surface tends to impair the adhesion of applied layers. To improve coating adhesion, Zr surfaces are currently roughened to achieve a moderately textured surface that facilitates improved bone-to-implant contact [21]. Nevertheless, an ideal surface topography that maximizes osteoblast attachment has yet to be definitively established [22].

The aim of our research is to enhance the osseointegration of zirconia dental implants through the application of a calcium phosphate-hydroxyapatite (CaP/HA) surface coating. Hydroxyapatite (HA) is an inorganic material with good biocompatibility and bone-conducting properties similar to those of natural bone, which promote the formation of new bone from the host bone along the implant interface or from inside the implant. HA coatings exhibit favorable physico-chemical properties that can synergistically regulate osteo-immunomodulation, bone/angiogenesis, and the interaction between immune regulation, osteogenesis, and angiogenesis to significantly improve osseointegration, thus offering an optimal strategy for the surface coating of biomedical implants [23, 24]. HA coatings also have a bone-strengthening effect on Zr [24]. HA can release calcium, which may react with zirconium oxide (ZrO) and lead to the formation of cubic ZrO, thereby inhibiting the hardening process [24, 25]. CaP coatings have also gained significant biological benefits in vivo and in vitro [26]. In vivo animal studies showed significantly higher bone to implant contact (BIC) values for CaP-coated Zr compared to non-coated implants, which achieve similar values to Ti [27]. Morphology has a strong influence on the results, as porous or nanostructured CaP coatings promote early bone apposition and vascular infiltration [28, 29].

In this study, we modified Zr discs with various coatings (Ti, CaP, HA and CaP/HA), evaluated their cytocompatibility in vitro with direct and indirect testings and investigated the surface structure and composition using scanning electron microscopy (SEM) combined with energy-dispersive X-ray spectroscopy (EDS). An osteoblast and a mouse-fibroblast cell-line were used to test either soft tissue response or verify improved bone formation.

## Results

### Cytocompatibility tests

#### Live/dead staining

**Fig. 1:**
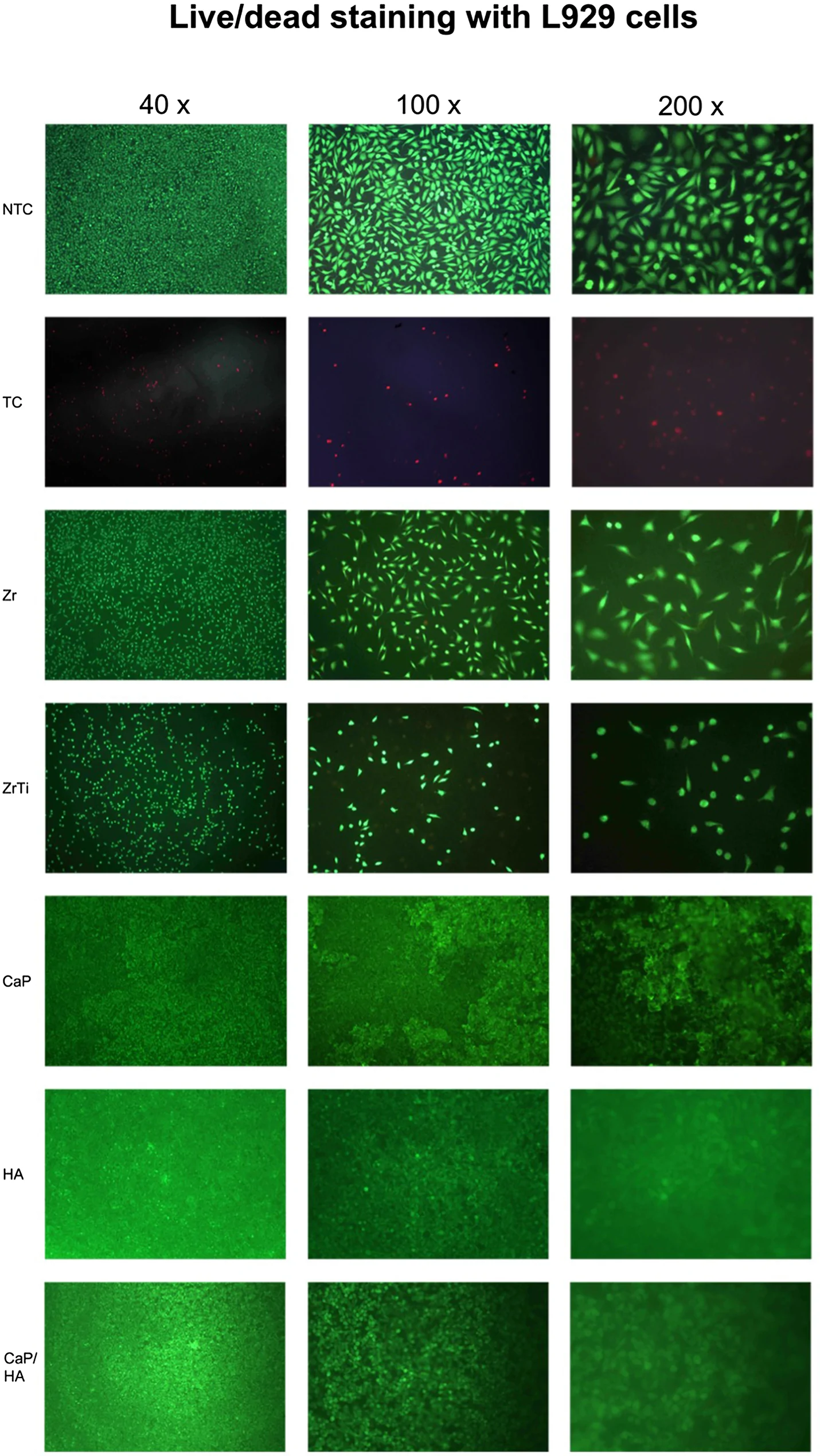
Results of the cytotoxicity testing with L929-mouse fibroblasts (live/dead staining) in direct contact with the different coating surfaces at different magnifications (40 x,100 x and 200 x)

**Fig. 2:**
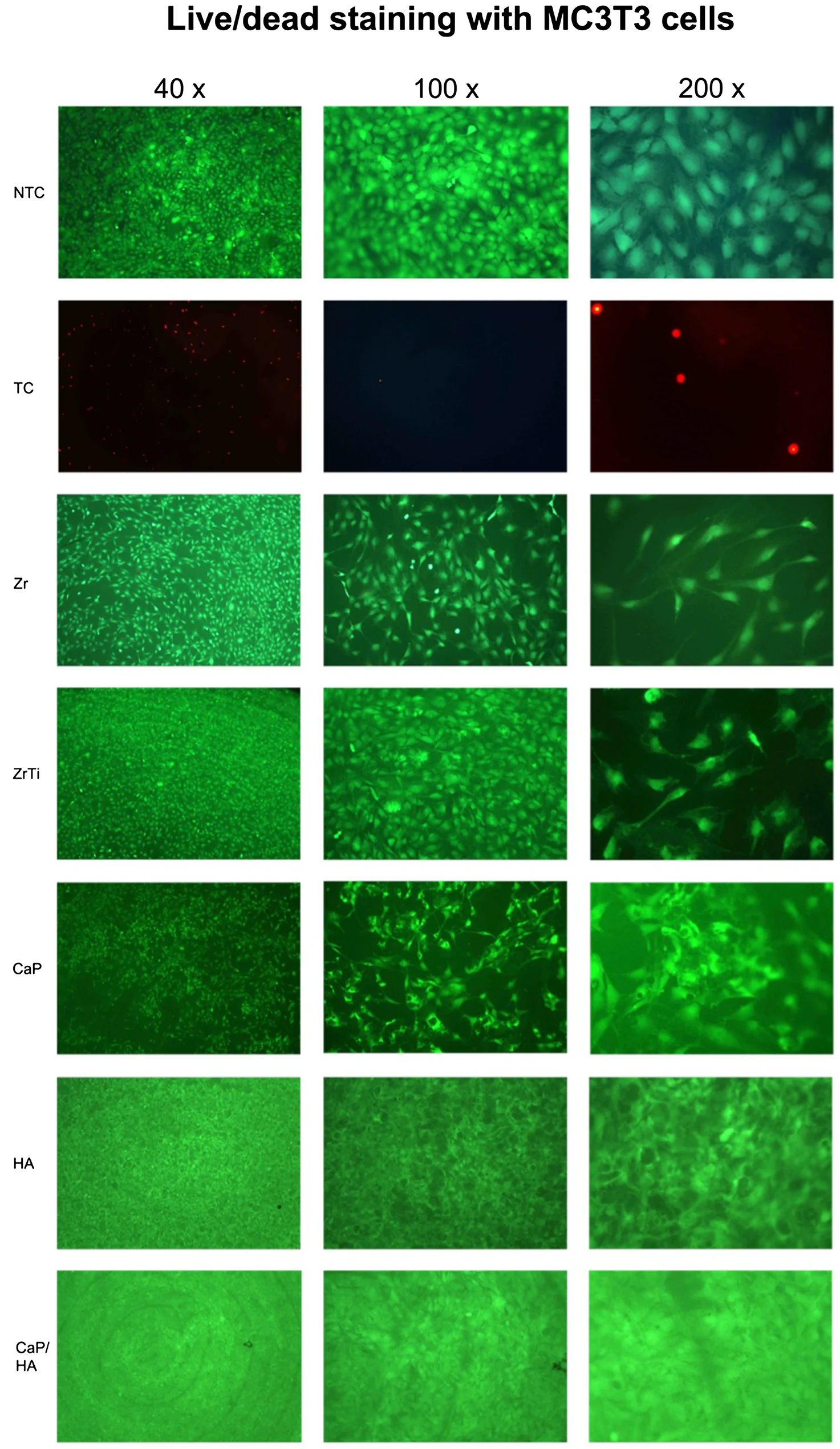
Results of the cytotoxicity testing with MC3T3 osteoblasts (live/dead staining) in direct contact with the different coating surfaces at different magnifications (40 x, 100 x and 200 x)

Live/dead staining with L929 fibroblasts and MC3T3 osteoblastic progenitor cells revealed numerous viable (Fluorescein Diacetate-positive) cells in all samples except the toxic control (TC), with no dead (Propidium Iodide-positive) cells observed (Figs. 1 and 2). Well-spread, spindle-shaped cells were particularly evident on the native Zr sample and present on other samples, except the toxic control. These results confirm that all six samples are cytocompatible and exhibit no cytotoxic effects.

#### LDH and XTT assay

The LDH assay was used to evaluate the cytotoxicity of various surface coatings in two cell lines: L929 mouse fibroblasts and MC3T3 osteoblastic progenitor cells. For both cell types, a one-way analysis of variance (ANOVA) revealed a statistically significant effect of the coating condition on LDH release (L929: F [6, 49] = 385.68, *p* <.001; MC3T3: F [6, 49] = 1467.02, *p* <.001), indicating that the tested surfaces had a measurable impact on cell membrane integrity.

**Fig. 3:**
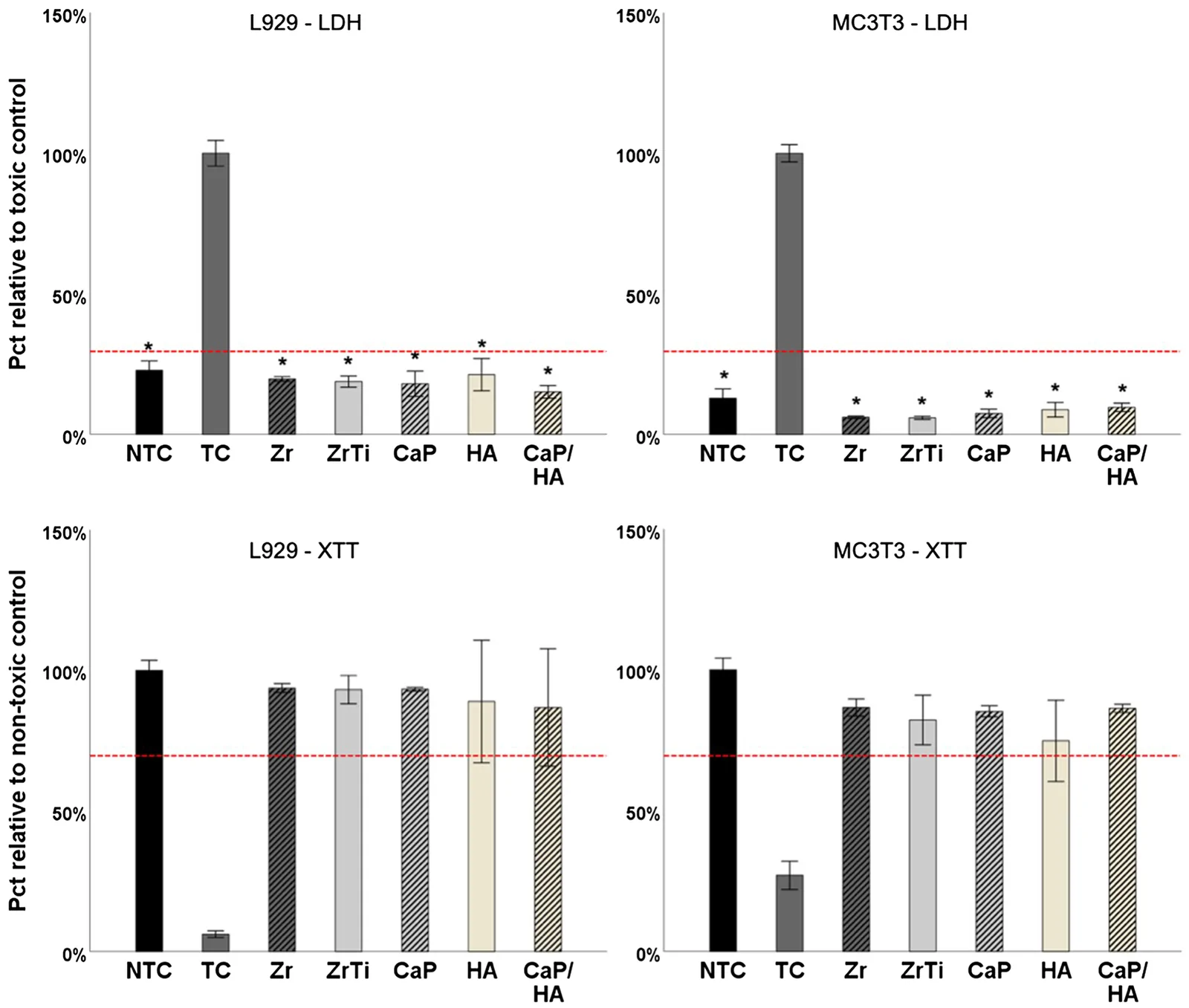
Results of the cytotoxicity testing with L929-mouse fibroblasts and MC3T3 osteoblasts (LDH- and XTT-assays) in indirect contact with the different coating surfaces (* *P* < .05; red line indicating 30% in LDH-assay and 70% in XTT-assay)

In L929 fibroblasts, the toxic control showed an LDH release of 100.0% (95% CI: 96.87–103.13), consistent with its role as the positive cytotoxic benchmark. In contrast, all coating conditions exhibited substantially lower LDH levels. Specifically, LDH release was 19.70% (95% CI: 16.56–22.83) for Zr, 18.79% (95% CI: 15.65–21.92) for ZrTi, 17.97% (95% CI: 14.84–21.11) for CaP, 21.22% (95% CI: 18.09–24.35) for HA, and 15.10% (95% CI: 11.96–18.23) for the combined CaP/HA coating. The non-toxic control (NTC) yielded a value of 22.79% (95% CI: 19.66–25.93). Post-hoc analysis confirmed that all coating conditions differed significantly from the toxic control (*p* <.001 in all comparisons). Moreover, LDH values remained well below 30% for all coatings, suggesting low cytotoxicity and good biocompatibility in this cell model (Fig. 3).

In MC3T3 osteoblastic progenitor cells, a similar pattern was observed. The toxic control again produced an LDH release of 100.0% (95% CI: 98.18–101.82), while all coated surfaces showed markedly reduced values. Zr and ZrTi coatings resulted in LDH releases of 6.12% (95% CI: 4.30–7.94) and 5.91% (95% CI: 4.09–7.73), respectively. CaP-coated surfaces showed 7.48% (95% CI: 5.67–9.30), HA coatings yielded 8.80% (95% CI: 6.98–10.61), and the CaP/HA combination resulted in 9.63% (95% CI: 7.81–11.45). The non-toxic control group showed a value of 12.92% (95% CI: 11.10–14.74). All coatings were significantly different from the toxic control in post-hoc comparisons (*p* <.001), and, critically, LDH release remained well below 30% across all surfaces. These findings indicate excellent cytocompatibility of the tested coatings in MC3T3 cells as well (Fig. 3).

Consistent with the LDH results, the XTT assay confirmed preserved metabolic activity in both cell models. In L929 fibroblasts, one-way ANOVA revealed a significant effect of the coating condition on XTT conversion (F [6, 49] = 44.50, *p* <.001). The non-toxic control (NTC) exhibited a mean value of 100.0% (95% CI: 90.08–109.92). All coatings demonstrated similarly high levels of metabolic activity, with no substantial deviations from the NTC. Zr, ZrTi, and CaP coatings each yielded values around 93.2–93.7%, while HA reached 88.9% (95% CI: 67.12–110.71) and CaP/HA showed 86.8 (95% CI: 65.92-107.71). Post-hoc testing confirmed that none of these coatings differed significantly from the NTC (*p* >.05), indicating that the metabolic activity of the cells remained unimpaired under all tested conditions (Fig. 3).

The findings were corroborated in MC3T3 osteoblastic progenitor cells, where ANOVA also revealed a significant coating effect (F [6, 49] = 62.78, *p* <.001). Here, the NTC again served as the benchmark (100.0%, 95% CI: 94.05–105.95). The tested coatings showed metabolic activity values between 74.8% and 86.6%. In L929 cells, none of the coatings differed significantly from the non-toxic control, indicating no detectable metabolic impairment in this cell line. In contrast, all coatings exhibited significantly reduced metabolic activity in MC3T3 cells when compared to the non-toxic control. Among these, Zr (86.6%; 95% CI: 83.53–89.69), ZrTi (82.2%; 95% CI: 73.37–90.96), CaP (85.2%; 95% CI: 83.27–87.22) and CaP/HA (86.3%; 95% CI: 84.84–87.72) showed comparatively higher values, whereas the HA coating resulted in the lowest metabolic activity (74.8%, 95% CI: 60.35–89.18). Overall, the results indicate a cell line–dependent reduction in metabolic activity, which should be considered when interpreting cytocompatibility from a metabolic perspective (Fig. 3).

### Surface morphology

**Fig. 4:**
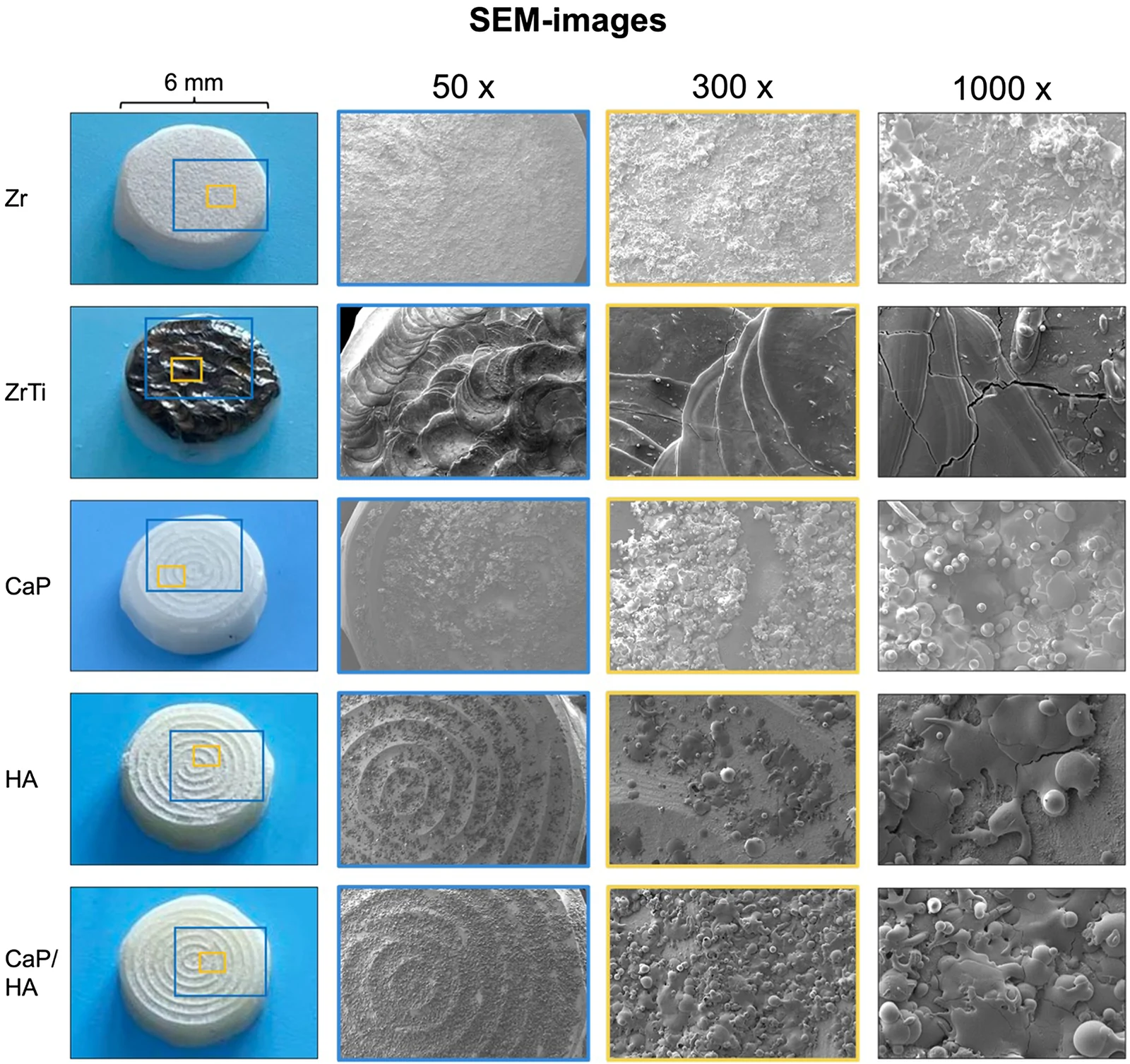
SEM-images of the different coating surfaces at different magnifications (50 x, 300 x and 1000 x)

All surfaces were successfully imaged using SEM, allowing for detailed visualization of surface roughness (Fig. 4). Where present, coatings were also clearly identifiable. The surface composition was further analyzed using EDS, which confirmed both the surface characteristics and the successful deposition of the various coating materials.

Due to the inherently smooth surface of Zr, mechanical interlocking is not feasible. As a result, adhesion relies heavily on thermal activation and the resulting surface structure. Our preliminary results indicate that bonding is fundamentally achievable, however, it is currently suboptimal and exhibits a strong dependency on the substrate properties.

#### Uncoated zirconia (Zr)

The native, uncoated Zr surface exhibited a visibly roughened topography under SEM, characterized by an irregular, microtextured structure. No surface coatings or modifications were observed, and the morphology was consistent across all examined areas.

**Fig. 5:**
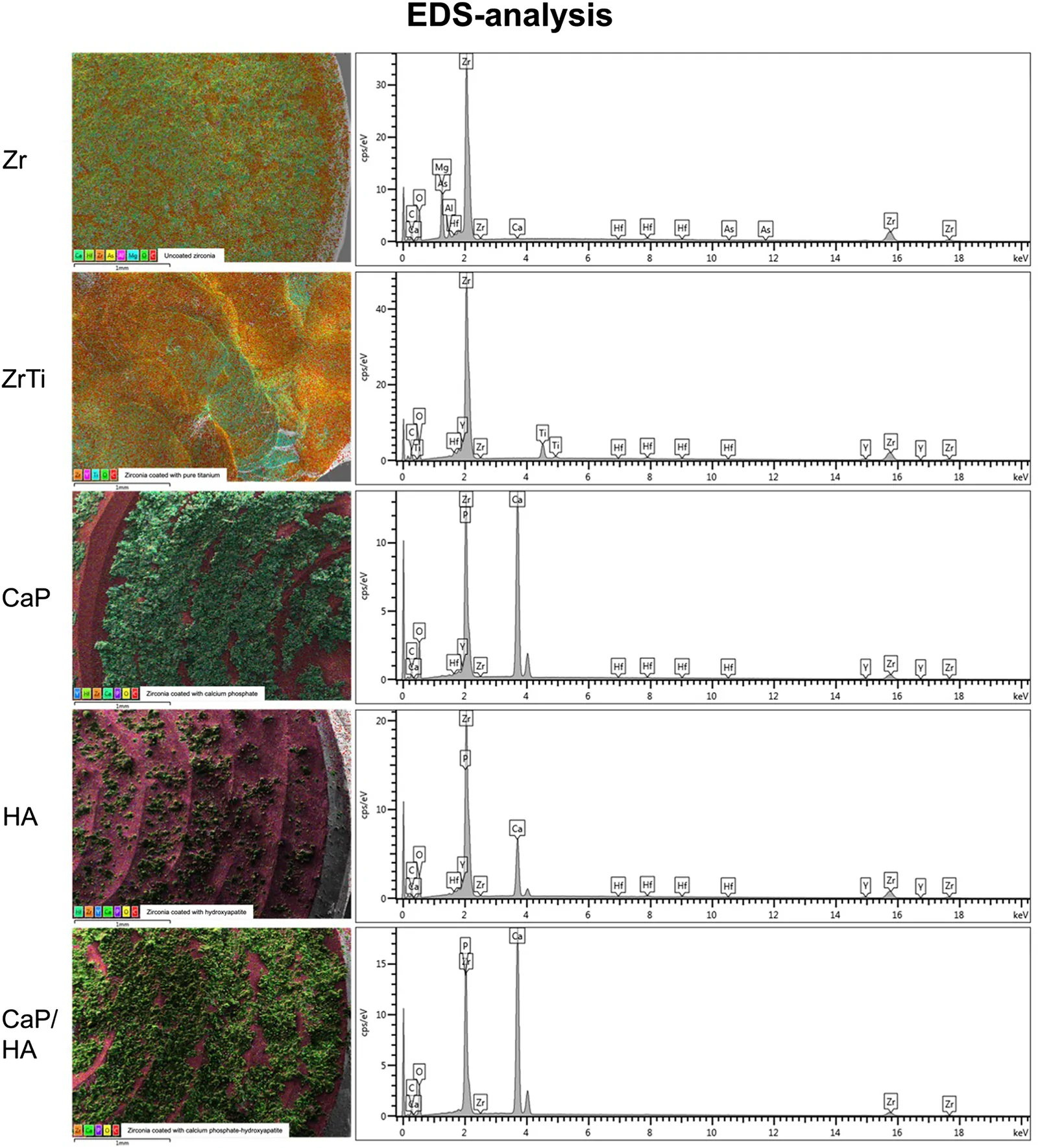
EDS-analysis of the different coating surfaces showing the surface characteristics (left) and the composition of the various coating materials (right)

EDS analysis confirmed a high content of Zr and oxygen (O), corresponding to the Zr substrate. Additionally, magnesium (Mg), arsenic (As), and calcium (Ca) were detected and found to be homogeneously distributed across the surface. Trace amounts of aluminum (Al) and hafnium (Hf) were also identified, though these elements appeared in a more localized, sporadic manner (Fig. 5).

#### Titanium-coated (ZrTi)

At 300× and 1000× magnification, the Ti-coated Zr surface exhibited visible microcracks. Spot analysis detected high levels of Zr and O, corresponding to the underlying Zr substrate, along with comparatively lower amounts of Ti. These findings suggest partial surface coverage or limited coating thickness in some areas (Fig. 5).

#### Calcium phosphate-coated (CaP)

SEM confirmed the presence of a successfully applied surface coating on the Zr substrate. The coating appeared as a distinct layer with varying thickness across the surface. It was primarily concentrated within the grooves of the surface topography, where it formed continuous deposits, while significantly less material was observed on the elevated ridges between grooves. This non-uniform distribution was consistently observed across all inspected areas.

EDS spot analysis further characterized the elemental composition of the coating. High levels of Ca and P were detected, consistent with the applied coating material, in addition to Zr and O signals originating from the underlying Zr substrate.

These findings indicate a heterogeneous surface coverage, with preferential deposition of the coating material in recessed features of the substrate’s surface (Fig. 5).

#### Hydroxyapatite-coated (HA)

The HA-coated specimen exhibited an accumulation of the coating material within the depths of the surface grooves as observed by SEM, while the edges and ridges between the grooves remained largely uncoated. EDS analysis revealed high levels of Zr and O from the underlying substrate, along with significant amounts of Ca and P, characteristic of the HA coating. Trace elements such as yttrium (Y) and Hf were also detected in low concentrations (Fig. 5).

#### Calcium phosphate and hydroxyapatite-coated (CaP/HA)

The specimens coated with calcium phosphate and hydroxyapatite displayed a relatively homogeneous coating pattern when examined by SEM. The coating was clearly visible both within the grooves and on the edges of the surface topography, indicating effective coverage across most regions of the samples. However, occasional uncoated spots were detected, predominantly located along the ridge areas between the grooves, suggesting slight inconsistencies in coating thickness or adherence in these elevated regions.

EDS analysis revealed pronounced signals for Ca and P, which are characteristic elements of the applied coatings. In contrast, the Zr and O signals, corresponding to the Zr substrate, were markedly reduced in intensity. This reduction indicates that the coating was sufficiently thick and uniform to obscure most of the underlying substrate from detection by EDS, confirming dense and comprehensive surface coverage (Fig. 5).

## Discussion

Traditionally, titanium implants, whether uncoated or surface-modified, are widely recognized for their osseointegrative capabilities and have been considered the gold standard for achieving successful osseointegration [30]. While surface modification agents, such as CaP and HA have been extensively studied on titanium dental implants, there remains a paucity of data regarding their application to zirconia dental implants [31–36]. The present study aimed to thoroughly evaluate the coating process and adhesion as well as the cytocompatibility of various surface coatings applied to Zr, targeting improved biological integration in both hard and soft tissues.

In this study, zirconia discs were coated with CaP and HA using atmospheric plasma spraying, and with Ti using wire-based laser metal deposition. Because insufficient adhesion often arises from mismatched thermal expansion coefficients between the substrate and the coating, several previous investigations have proposed the use of intermediate interfacial layers to enhance bonding [37, 38]. By employing a modified atmospheric plasma spraying protocol that incorporated substrate preheating—an approach not previously described for coating zirconia implants—we achieved satisfactory adhesion of CaP, HA, and combined CaP/HA layers.

The primary rationale for applying a Ti coating onto zirconia is to obtain an implant with a zirconia core which remains still suitable for electrolysis-based biofilm removal, a process driven by the passage of electric current [39]. Although plasma-sprayed Ti coatings on zirconia have been previously attempted by Si et al., the reported adhesion was suboptimal [40]. Therefore, we evaluated laser metal deposition as an alternative Ti-coating method. One major challenge encountered during Ti deposition on Zr was excessive heat generation, resulting in a 40% fracture rate of the Zr specimens. Additionally, laser metal deposition produced coatings with substantial surface irregularities, inconsistent thickness and visible cracking, as illustrated in Fig. 4. Other approaches, such as sol-gel-derived Ti coatings, have likewise yielded discontinuous and uneven layers [41]. A recently reported and promising technique for producing porous titania-coated zirconia by Tang et al. involves immersing zirconia substrates in zirconium oxychloride and titanium oxide solutions [42]. The parameters of titanium surface modification should be reevaluated, as the release of material into the surrounding soft tissue may lead to dehiscence and inflammation. Therefore, this group will not be included in further research projects. Further investigations may profitably explore sputtering of Ti onto zirconia implants under vacuum conditions, as described by Kimura et al., who applied a thin Ti layer and achieved strong shear bond performance [43].

In the present study, coating adhesion was not quantitatively assessed by scratch testing according to ISO 15,184. Instead, mechanical adhesion between the substrate and coating was evaluated qualitatively by visual inspection. All coatings remained intact during sample transport and preparation for SEM and EDS analyses, indicating adequate adhesion. Indeed, all surfaces demonstrated coherent coating integrity and bonding, parameters essential for long-term stability and biological performance in clinical applications (Figs. 4 and 5).

Zr, as a biomaterial, has gained more and more attention in dental implantology due to its favorable mechanical and aesthetic characteristics [13]. However, our findings are consistent with previously published studies, which emphasize that additional surface modifications, especially concerning the topographical and chemical surface composition, are necessary to improve bioactivity [44]. Regarding dental implants, matching osteoblast and fibroblast responses are particularly noteworthy, as osteoblastic activity is necessary for osseointegration and fibroblast function ensures proper soft tissue sealing. In vitro it is not yet possible to fully imitate the complex biological system. In this study, MC3T3 cells were employed as a well-established murine pre-osteoblastic cell line that serves as a reliable model for human osteoblasts in vitro. Furthermore this cell line is often used in dental implant research, facilitating comparative analysis across related studies [45]. L929 mouse fibroblasts were applied to simulate the adhesion of the soft tissue sealing. In this study, all modified surfaces demonstrated cytocompatibility in assessments using MC3T3 pre-osteoblasts and L929 fibroblasts, suggesting their potential to support both effective osseointegration and soft tissue integration in clinical applications. In future studies, other cell lines, such as human gingival fibroblasts (HGFs), could be used to provide more tissue-specific insights.

Across all tested materials, including uncoated Zr, coatings of pure Ti, CaP, HA and a combination of CaP and HA, no cytotoxic effects were observed in both direct (24 h live/dead staining) and indirect (72 h XTT and LDH assays) assessments. All surfaces showed robust cell viability and adhesion. In particular, the CaP/HA coating showed no negative effects and tended to achieve equally good or better values (e.g., minimally lowest LDH release) than the other surfaces. These findings are in accordance with current trends in implantology, highlighting the importance of optimizing surface characteristics to increase cellular response and tissue compatibility and integration [46, 47].

Although a statistically significant reduction was only observed in the MC3T3 LDH assay, HA consistently showed a more pronounced deviation in all other experiments compared with the other coatings. As suggested in previous studies, the nanostructure of the coating may influence bioactivity. Specifically, crystalline HA layers obtained through post-deposition annealing at 400–600 °C have been associated with enhanced biocompatibility [48–50]. It should be noted, however, that these findings were derived from coatings on Ti rather than on Zr substrates. Alternative strategies to generate HA top layers on Zr include plasma electrolytic oxidation (PEO) [51]. Cengiz et al. reported improvements using single-step PEO, whereas Yan et al. achieved superior results by applying additional acidic or alkaline surface activation following PEO [52, 53]. Further research is warranted to optimize these coating approaches.

According to the direct and indirect cytocompatibility assessments conducted here, demonstrating stable metabolic activity across all samples, previous reports corroborate on the compatibility of inorganic coatings with osteogenic cells as summarized in the meta-analysis by Jenny et al. [54]. This meta-analysis also showed a significant mean BIC increase (~ 14.7%) comparing inorganic coatings to controls [54]. It should be noted that only 2 of the 32 studies included evaluated zirconia dental implants, while the remaining studies focused exclusively on titanium implants [55, 56]. Another review showed that implant longevity and osseointegration improved with bioactive surface modifications - an average BIC increase of 7.29% and improved stability [57]. Although this review included only titanium implants, the findings emphasize the clinical relevance of the present results and support our conclusion that CaP and HA coatings drive meaningful biological responses.

Assuming that the osseointegration of zirconia implants can be enhanced through the application of alternative bioactive coatings, reliance on Ti may no longer be essential. This offers a compelling metal-free alternative, particularly beneficial for patients with Ti hypersensitivity or heightened aesthetic requirements. Moreover, the combination of inorganic coatings such as CaP with organic bioactive components - such as proteins or growth factors - should be explored, as previous work has demonstrated promising synergistic effects [58, 59].

It is essential to note that in vitro cytocompatibility tests are just an initial step [60]. Cell culture systems cannot fully replicate the complexity of the in vivo environment. The complexity of in vivo tissue responses includes among other aspects loading forces, inflammatory responses and long-term dynamics. This study provides a basis for subsequent investigations. Therefore, future studies should include long-term stress tests, preclinical animal models and randomized clinical trials to assess osseointegration, peri-implant soft tissue health, long-term stability and safety of these innovative coatings under physiological condition, particularly comparing coated to uncoated and Ti‐coated zirconia implants. In a further research approach, additional antibacterial additives could be mixed into the layer, such as silver or zinc nanoparticles, regarding Paiwand et al. 2025 [36].

## Conclusions

This study contributes valuable data supporting the biocompatibility of zirconia implants modified with calcium phosphate and hydroxyapatite-based coatings. These modifications hold potential for improving both soft and hard tissue integration, thus enhancing the clinical outcomes of ceramic dental implants. They also offer promising alternatives to titanium coatings, especially in metal-sensitive or aesthetic cases. Future research should focus on in vivo validation and long-term clinical performance.

## Methods

### Specimen production

A total of 40 ceramic discs (goodBIONICS Biotechnologie GmbH, Mauerstetten, Germany) with a diameter of 6 mm and height of 2 mm were milled from a ceramic block VITA YZ^®^ T (VITA Zahnfabrik H. Rauter GmbH & Co. KG, Bad Säckingen, Germany). Eight of these specimens were left in their native state without coating, while 32 underwent surface modification performed by a specialized coating company (Titan-Präcis Metallurgie GmbH, Henstedt-Ulzburg, Germany). Among the modified specimens, eight were coated with pure Ti, eight with CaP (EMPROVE^®^ ESSENTIAL, Merck KGaA, Darmstadt, Germany), eight with HA (Sigma‑Aldrich Hydroxyapatite, Merck KGaA, Darmstadt, Germany) and eight with CaP/HA (*n* = 8). For each experiment *n* = 8 for each group were tested.

Initially, the specimens were thoroughly degreased to remove organic contaminants. Based on prior findings in preliminary testing indicating that conventional grit blasting with alumina, typically employed to increase surface roughness, paradoxically leads to surface smoothing in Zr, this step was entirely omitted. Instead, surface activation was performed directly using a plasma flame. This approach enhances adhesion by simultaneously cleaning the surface and applying targeted thermal treatment.

The ceramic specimens were coated with Ti using wire-based laser metal deposition. The coating process was performed under argon shielding to prevent oxidation during laser treatment. Owing to the pronounced differences in thermophysical properties between zirconia and titanium, no melt intermixing or metallurgical bonding occurs at the interface. Instead, the titanium is briefly molten in the laser focal zone just above the ceramic surface and subsequently deposited in its liquid state. Upon cooling, the titanium layer contracts and thereby forms a predominantly mechanical joint with the zirconia substrate.

For coating the ceramic discs with CaP, HA and CaP/HA, atmospheric plasma spaying was used. The specimens were mounted in a specialized holder and placed into the plasma chamber. Upon initiation of the process, the plasma flame was first operated without powder injection to thermally activate the surface and remove residual contaminants. The plasma temperature in the flame core ranges from 10,000 to 20,000 K, depending on the measurement location. However, due to the steep thermal gradient, the surface temperature of the specimen remained below approximately 200 °C, depending on the exposure duration.

Following the activation phase, the respective coating powder (CaP, HA and CaP/HA) was introduced into the plasma flame via an argon carrier gas. The powder was partially melted in the plasma and propelled toward the surface at high velocity, where it adhered due to adhesive interactions.

Upon completion of the coating process, the surface was flushed with pure argon gas to remove loose particles and residues, thereby preparing it for subsequent analyses. Coated surfaces were examined using a Keyence VHX-7000 digital microscope (Keyence Corporation, Osaka, Japan). This includes assessment of layer homogeneity, surface topography and documentation of potential cracks or delamination.

### Cytocompatibility assessment

#### Cell cultivation and reference materials

L929 mouse fibroblasts and MC3T3 osteoblast progenitor cells were obtained from Sigma-Aldrich (Sigma–Aldrich, St. Louis, USA). The cells were cultured in Minimum Essential Medium (MEM) supplemented with 10% fetal bovine serum (FBS), 100 U/mL penicillin, 100 µg/mL streptomycin (all from Life Technologies, Carlsbad, USA), and L-glutamine (Sigma–Aldrich, St. Louis, USA) to a final concentration of 4 mM. Cells were maintained in 12-well plates at 37 °C in a humidified incubator with 5% CO₂ and 95% relative humidity. Once cultures reached approximately 80% confluence, cells were passaged. Reference Material A (RM-A; Hatano Research Institute, Food and Drug Safety Center, Japan) was employed as a toxic control (TC), while tissue culture coverslips (TCC; Sarstedt, Nümbrecht, Germany) served as a non-toxic control (NTC).

#### Live/dead staining assay

A live/dead staining assay with L929 fibroblasts and MC3T3 osteoblast progenitor cells was used to evaluate cytocompatibility. Samples were directly seeded with L929 and MC3T3 cells in cell culture medium at a surface-to-volume ratio of 5.65 cm²/mL in 12-well plates. After 24 h of incubation under standard cell culture conditions, 60 µL/mL of propidium iodide stock solution (50 µg/mL in PBS) and 500 µL/mL of fluorescein diacetate (FDA) working solution (20 µg/mL in PBS, prepared from a 5 mg/mL FDA stock in acetone) were added to each well. Following a short incubation period of 3 min at room temperature, the samples were rinsed with PBS and immediately examined using an upright fluorescence microscope (Nikon ECLIPSE Ti–S/L100, Nikon, Düsseldorf, Germany).

#### Lactate dehydrogenase (LDH) and cell proliferation (XTT) assay

Cytocompatibility testing was carried out in accordance with DIN EN ISO 10993-5/-12.

For the preparation, disinfected test specimens were transferred into 12-well plates and overlaid with 1.88 mL of cell culture medium per well (equivalent to 3 cm²/mL). The plates were incubated for 72 h at 37 °C in a humidified atmosphere with 5% CO₂. Following incubation, the supernatant was centrifuged and the resulting clarified extract was carefully transferred into a new tube.

Positive control extracts were prepared using RM-A discs with a surface area equivalent to that of the test specimens, incubated in 1.88 mL of cell culture medium (3 cm²/mL). TCC in 13 mm diameter served as the negative control.

After the extraction period, the resulting extracts were applied to prepared cells (L929 and MC3T3) and incubated for 24 h under standard culture conditions (37 °C, 5% CO₂). Microscopic evaluation was then performed.

For the lactate dehydrogenase (LDH) assay, a LDH reaction mix was prepared by combining 200 µL of WST Substrate mix with 10 mL of LDH assay buffer. From this mix, 100 µL was added to 10 µL of cell culture supernatant in each well and incubated for 30 min at room temperature. Optical density was measured at 450 nm with a reference wavelength of 650 nm using the SPECTROstar Omega microplate reader (BMG Labtech, Ortenberg, Germany).

For the cell proliferation (XTT) assay, the labeling mixture was prepared by mixing 0.1 mL of electron-coupling reagent with 5 mL of XTT labeling reagent. 50 µL of this mixture was added to each well and incubated for 4 h at 37 °C in a 5% CO₂ incubator. The optical density was then measured directly—without transferring the supernatant to a new plate—at 450 nm and 650 nm using the same microplate reader.

### Surface morphology analysis

The surface morphology and elemental composition of the specimens were analyzed using scanning electron microscopy (SEM; Crossbeam 340, Zeiss, Oberkochen, Germany) equipped with an energy-dispersive X-ray spectroscopy system (EDS; X-Max Silicon Drift Detector, Oxford Instruments, Abingdon, UK). Data acquisition and evaluation were performed with AZtec software (version 3.3, Oxford Instruments). Specimens were mounted on aluminum stubs and sputter-coated with carbon to ensure conductivity. Accordingly, the carbon peak was excluded from elemental analysis. SEM images were obtained in secondary electron (SE) mode at magnifications of 50× to 1,000×, with a working distance of 5 mm and an accelerating voltage of 5–30 kV. For EDS, area scans of representative surface regions were acquired until approximately one million counts were reached to ensure sufficient statistical accuracy. Elemental compositions were obtained by semi-quantitative, standardless analysis with ZAF correction using the AZtec software.

### Statistical analysis

All statistical analyses were performed using IBM SPSS Statistics (IBM, New York, USA). For each assay (LDH and XTT), data were first inspected descriptively by calculating group-wise means, standard errors, and 95% confidence intervals. To assess the effects of surface coatings on cell viability and cytotoxicity, one-way analyses of variance (ANOVA) were conducted separately for each cell type (L929 and MC3T3) and for each assay.

In the inferential analysis, surface coating was treated as a fixed factor with seven levels (NTC, TC, Zr, ZrTi, CaP, HA, CaP/HA). The primary outcome variables were LDH release and XTT metabolic activity, expressed as percentage values normalized to the appropriate control group (TC for LDH; NTC for XTT). Following significant omnibus tests, estimated marginal means were computed to describe group effects while adjusting for within-group variability. Post hoc comparisons between individual coating conditions and control groups were performed using Bonferroni correction to adjust for multiple testing. Significance was defined as *p* < .05 in all tests. Confidence intervals for the estimated marginal means were set at the 95% level and reported alongside means to convey the precision of the estimates.

## Data Availability

The datasets generated and/or analysed during the current study are available from the corresponding author on reasonable request.

## Abbreviations

Al: Aluminium
ANOVA: Analysis of variance
As: Arsenic
BIC: Bone to implant contact
Ca: Calcium
CaP: Calcium phosphate
CaP/HA: Calcium phosphate-hydroxyapatite
EDS: Energy-dispersive X-ray spectroscopy
HA: Hydroxyapatite
Hf: Hafnium
LDH: Lactate dehydrogenase
Mg: Magnesium
NTC: Non-toxic control
O: Oxygen
PEO: Plasma electrolytic oxidation
SEM: Scanning electron microscopy
Ti: Titanium
XTT: 2,3-bis-(2-methoxy-4-nitro-5-sulfophenyl)-2 H-tetrazolium-5-carboxanilide
Y: Yttrium
Zr: Zirconia
ZrO: Zirconium oxide
ZrTi: Zirconia coated with pure titanium
